# Development and Preclinical Evaluation of Fixed-Dose Capsules Containing Nicergoline, Piracetam, and Hawthorn Extract for Sensorineural Hearing Loss

**DOI:** 10.3390/pharmaceutics17081017

**Published:** 2025-08-05

**Authors:** Lucia Maria Rus, Andrei Uncu, Sergiu Parii, Alina Uifălean, Simona Codruța Hegheș, Cristina Adela Iuga, Ioan Tomuță, Ecaterina Mazur, Diana Șepeli, Irina Kacso, Fliur Macaev, Vladimir Valica, Livia Uncu

**Affiliations:** 1 Department of Pharmaceutical Analysis, Faculty of Pharmacy, “Iuliu Hațieganu” University of Medicine and Pharmacy, 400349 Cluj-Napoca, Romania; lucia.rus@umfcluj.ro (L.M.R.); alina.uifalean@umfcluj.ro (A.U.); cmaier@umfcluj.ro (S.C.H.); cristina.iuga@medfuture.ro (C.A.I.); 2 Scientific Center of Medicines, “Nicolae Testemițanu” State University of Medicine and Pharmacy of Republic of Moldova, MD-2025 Chisinau, Moldova; andreiuncu1990@gmail.com (A.U.); sergiu.parii@usmf.md (S.P.); ecaterina.mazur@usmf.md (E.M.); vladimir.valica@usmf.md (V.V.); livia.uncu@usmf.md (L.U.); 3 Department of Personalized Medicine and Rare Diseases, MEDFUTURE—Institute for Biomedical Research, “Iuliu Hațieganu” University of Medicine and Pharmacy, 400347 Cluj-Napoca, Romania; 4 Department of Pharmaceutical Technology and Biopharmacy, Faculty of Pharmacy, “Iuliu Hațieganu” University of Medicine and Pharmacy, 400012 Cluj-Napoca, Romania; tomutaioan@umfcluj.ro; 5 Department of Pharmaceutical and Toxicological Chemistry, “Nicolae Testemițanu” State University of Medicine and Pharmacy of Republic of Moldova, MD-2025 Chisinau, Moldova; 6 Institute of Chemistry, State University of Moldova, MD-2028 Chisinau, Moldova; dianashepel@mail.ru (D.Ș.); fliur.macaev@ichem.md (F.M.); 7 National Institute for Research and Development of Isotopic and Molecular Technologies, 400293 Cluj-Napoca, Romania

**Keywords:** fixed-dose combinations, nicergoline, piracetam, hawthorn extract, capsule formulation, preformulation, antioxidant assay

## Abstract

**Background:** Fixed-dose combinations have advanced in many therapeutic areas, including otorhinolaryngology, where hearing disorders are increasingly prevalent. **Objectives:** The present study focuses on developing and evaluating a new capsule combining nicergoline (NIC), piracetam (PIR), and hawthorn extract (HE) for the management of sensorineural hearing loss. **Methods:** The first phase methodology comprised preformulation studies (DSC, FTIR, and PXRD) to assess compatibility among active substances and excipients. Subsequently, four formulations were prepared and tested for flowability, dissolution behavior in acidic and neutral media, and stability under oxidative, thermal, and photolytic stress. Quantification of the active substances and flavonoids was performed using validated spectrophotometric and HPLC-UV methods. **Results:** Among the tested variants, the F1 formulation (4.5 mg NIC, 200 mg PIR, 50 mg HE, 2.5 mg magnesium stearate, 2.5 mg sodium starch glycolate, and 240.5 mg monohydrate lactose per capsule) displayed optimal technological properties, superior dissolution in acidic media, and was further selected for evaluation. The antioxidant activity of the formulation was confirmed through the 2,2-diphenyl-1-picrylhydrazyl (DPPH) assay, Trolox Equivalent Antioxidant Capacity (TEAC), and iron chelation tests, and was primarily attributed to the flavonoid content of the HE. Acute toxicity tests in mice and rats indicated a high safety margin (LD_50_ > 2500 mg/kg), while ototoxicity assessments showed no adverse effects on auditory function. **Conclusions:** The developed formulation displayed good stability, safety, and therapeutic potential, while the applied workflow could represent a model for the development of future fixed-dose combinations.

## 1. Introduction

Fixed-dose combinations account for approximately one-third of the medications used in general practice and one-fifth in hospital therapy [1]. Recently, fixed-dose combinations have made significant advances across various therapeutic areas, and their use continues to expand, including in otorhinolaryngology, where the prevalence of hearing disorders is on the rise [2,3,4]. Deafness and hearing loss represent a global issue, affecting over 1.5 billion people, according to the WHO, with this number estimated to reach 2.5 billion by 2050 [5].

The treatment of sensorineural hearing loss is usually symptomatic and includes vasodilators, anti-inflammatories, nootropics, and antioxidants [6,7]. Considering the complex etiology and multifactorial pathophysiology of this condition, the development of combination therapies is of growing interest [8].

Nevertheless, the development of such combined medications is a complex process, requiring compatibility studies between active ingredients and excipients, carried out using complementary techniques such as Differential Scanning Calorimetry (DSC), Fourier Transformed Infrared Spectroscopy (FTIR), and X-ray Powder Diffraction (XRPD) [9]. In capsule development, the selection of appropriate excipients is essential, influencing pharmaco-technical parameters and the dissolution profile, which is an important indicator of bioavailability [9,10]. Given the extensive use and significance of fixed-dose combinations in modern treatments, the development of efficient and reliable analytical methods that can simultaneously determine substances with various chemical structures represents a significant challenge for analytical experts [11]. In this regard, by applying accurate, sensitive, reproducible, accessible, and simple methods such as UV-VIS spectrophotometry and high-performance liquid chromatography (HPLC), the simultaneous determination of components in combinations can be ensured, including in stability evaluation [12,13,14].

In the present study, a novel fixed-dose combination targeting sensorineural hearing loss was developed, based on the hypothesis of vascular involvement in its pathogenesis. The formulation combines nicergoline (NIC), piracetam (PIR), and a plant-derived antioxidant, hawthorn extract (HE). This combination was selected based on solid evidence from the literature regarding their physicochemical compatibility, favorable safety profiles, and, not least, their therapeutic potential hearing-related disorders. 

**Figure 1:**
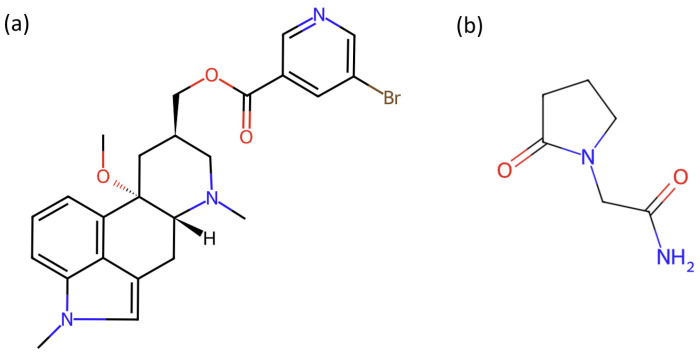
Chemical structures of (**a**) nicergoline and (**b**) piracetam.

Nicergoline (Figure 1a, [15]), a semisynthetic ergot derivative, is a white to off-white solid, insoluble in water (0.002 mg mL^−1^ at 25 °C) in its crystalline commercial form. It is stable between pH 4 and 9, but outside this range it degrades, primarily through oxidation, forming nicotinamide, *N*-methylnicotinamide, and *N*-acetylnicotinamide [16]. In a study designed to evaluate the effectiveness of NIC in preventing acoustic trauma, results revealed that NIC prevented noise-induced hearing loss and enhanced the hearing threshold [17]. These findings were later confirmed in a clinical trial, which demonstrated that NIC may attenuate a temporary threshold shift and self-reported tinnitus in military personnel exposed to noise [18]. The neuroprotective effects of NIC are attributed to its antioxidant and neurotrophic properties, as well as its ability to enhance inner ear blood flow [17].

Piracetam (Figure 1b, [15]), a cyclic derivative of gamma-aminobutyric acid (GABA), is a water-soluble crystalline compound that exhibits polymorphism. It shows stable behavior, even under acidic conditions, during neutral hydrolysis, under oxidative or thermal stress, and direct sunlight exposure. It was found to degrade only under alkaline conditions [19]. Similarly to NIC, PIR has proven beneficial effects on recovery of cochlear damage due to acoustic trauma on experimental animals using histopathologic and electrophysiologic examinations [20]. Used for its neuroprotective and antioxidant effects, PIR also exerts antithrombotic effects by inhibiting platelet aggregation, reducing fibrinogen and von Willebrand factor levels, and improving cerebral perfusion through decreased blood viscosity and vasospasm [20].

Hawthorn is a well-known medicinal plant, rich in flavonoids and proanthocyanidins, such as procyanidin B2, epicatechin, chlorogenic acid, hyperoside, and isoquercitrin [21]. It exhibits vasodilatory properties by stimulating nitric oxide release and thereby lowering peripheral vascular resistance. Additionally, its antioxidant properties support endothelium function by protecting vessels from oxidative stress [22].

In the acute phase of sensorineural hearing loss, treatment options are limited, with systemic corticosteroids being the standard approach, though their effectiveness remains limited. In the stable phase, management relies solely on medical devices such as hearing aids and cochlear implants [23]. The combination of vasodilators and antioxidant agents might represent a promising alternative to systemic corticosteroids or allow for reduced steroid doses. This synergistic approach has already demonstrated a superior efficacy compared to individual compounds, as shown in cisplatin-induced hearing loss [24] or age-related hearing loss [25]. Furthermore, a 2020 review concluded that a combination of specific antioxidants (such as *N*-acetylcysteine, HK-2, and vitamins A, C, and E) and the vasodilator Mg^2+^ may enhance protection against noise-induced damage through synergistic or complementary mechanisms [26].

Therefore, the development of fixed-dose capsules containing NIC, PIR, and HE are of great interest and may offer a more effective and safer therapeutic strategy for managing sensorineural hearing loss. By targeting both vascular and oxidative mechanisms implicated in cochlear damage, this combination could enhance cochlear perfusion, reduce oxidative stress, and support neuronal protection.

The main objective of this study was to investigate the physicochemical and pharmacological parameters relevant to the development of fixed-dose combination capsules containing PIR, NIC, and HE.

Compatibility between components was conducted using DSC, FTIR, and XRPD techniques. Four capsule formulations underwent preformulation optimization, pharmaco-technical evaluation, and dissolution testing. UV-VIS spectrophotometric and HPLC methods were developed and validated for the simultaneous analysis of NIC and PIR, while HE flavonoids were quantified by HPLC. Additionally, the capsules’ stability was investigated under various stress conditions (oxidative, photolytic, and thermal). Finally, antioxidant, acute toxicity, and ototoxicity activities of the final capsule formulation were evaluated.

Beyond addressing the specific formulation challenges of fixed-dose combination capsules, the study serves as a comprehensive model for the step-by-step development of a multicomponent pharmaceutical product, covering key formulation and evaluation phases.

## 2. Materials and Methods

### 2.1. Materials and Reagents

Nicergoline (NIC) from Delta Pharma (Cairo, Egypt), piracetam (PIR) from Northeast General Pharmaceutical Factory (Shenyang, China), and hawthorn extract (HE), derived from *Crataegus monogyna* and supplied by Euromed Group (Barcelona, Spain), were used as active pharmaceutical ingredients (APIs) in the capsule formulation development. The excipients used in capsule preparation included monohydrate lactose 200 mesh crystalline (ML, Pharmachemicals Handels GmbH, Hamburg, Germany), microcrystalline cellulose Avicel PH 102 (MC, JRS Pharma GmbH, Rosenberg, Germany), sodium starch glycolate (SSG, JRS Pharma GmbH, Rosenberg, Germany), magnesium stearate (MS, Stanchem Sp. J., Niemce, Poland), and polyvinylpyrrolidone K30 (PVP K30, ISP, Toms River, NJ, USA).

For the development and standardization of the analytical methods, reference standards of the APIs were obtained: NIC (Sigma-Aldrich, Taufkirchen, Germany), PIR (Merck, Darmstadt, Germany), and HE (Selleck Biotechnology GmbH, Cologne, Germany). In addition, rutoside trihydrate (Sigma-Aldrich, Taufkirchen, Germany) was used as the reference standard for flavonoid content determination. Three experimental batches of operculated capsules were prepared under laboratory conditions for all studies. All solvents used were of chromatographic purity grade, and reagents complied with the specifications of the European Pharmacopoeia.

### 2.2. Evaluation of API–Excipient Thermal Compatibility

Differential Scanning Calorimetry (DSC) was used to assess the thermal compatibility between the active pharmaceutical ingredients (NIC, PIR, and HE) and the selected excipients (ML, MC, SSG, MS, and PVP K30), including their binary physical mixtures (1:1, *w*/*w*). Approximately 2.00 ± 0.05 mg of each sample was accurately weighed into a 40 μL aluminum crucible and analyzed using a DSC 822 System (Mettler Toledo GmbH, Greifensee, Switzerland). The samples were heated from 25 °C to 400 °C at a rate of 10 °C/min under a nitrogen atmosphere (80 mL/min flow rate). The apparatus was calibrated using an indium standard placed in a 40 µL aluminum crucible. Data acquisition and analysis were conducted using STAR^e^ SW version 12.10 Software (Mettler Toledo GmbH, Giessen, Germany).

### 2.3. Detection of API–Excipient Interactions

Fourier Transform Infrared Spectroscopy (FTIR) was employed during the preformulation stage to investigate potential interactions between the active pharmaceutical ingredients (APIs), excipients, and their physical mixtures. Spectra were recorded using a Perkin Elmer Spectrum 100 FTIR spectrometer (PerkinElmer Inc., Waltham, MA, USA) in the 4000 to 650 cm^−1^ spectral range, with a resolution of 4 cm^−1^, using a KBr pellet technique. Each sample was dispersed in about 300 mg of anhydrous KBr and mixed in an agate mortar. The KBr pellet was obtained in an exhaust mold by pressing the ground mixture. 

### 2.4. Solid-State Characterization and Compatibility

Powder X-ray Diffraction (PXRD) was used to assess potential incompatibilities between the active ingredients and excipients during the preformulation step. Measurements were performed using a Bruker D8 Advance diffractometer equipped with a Ge (111) monochromator for CuKα1 radiation with λ = 1.54056 Å, operating at 40 kV and 40 mA. Patterns were recorded with a LynxEye superspeed detector in the 5–50° angular 2θ range, with a scan rate of 0.02°/s.

### 2.5. Evaluation of Powder Flow and Compressibility Parameters

To assess the flowability and compressibility of the powder blends, parameters which directly impact capsule filling efficiency and uniformity, four capsule formulations (F1–F4) were prepared by blending active substances with different excipients, as presented in Table 1.

Rheological properties, such as the angle of repose, the tilting angle, bulk and tapped densities, Carr’s Compressibility Index, and Hausner ratio [12], were determined using standard procedures and equipment (ERWEKA VP-12A, Pascall 545-P-AK-3).

The angle of repose was determined using the fixed-cone method. A quantity of 50 g of powder blend was loaded into the funnel of the VP-12A powder flow tester. When the shutter was opened, the powder flowed freely onto a horizontal surface, forming a conical heap. The angle between the slope of the cone and the horizontal plane was measured using a protractor. Five measurements were performed for each sample, and the average was calculated. This angle provides a widely accepted indirect measure of powder flowability; lower angles indicate better flow.

The tilting angle was determined as a complementary method. In this case, 50 g of powder was loaded into the VP-12A device, and after opening the shutter, the powder formed a cone on an inclined surface. The angle formed between the tangent to the slope of the powder cone and a horizontal reference line passing through the apex was measured. This technique is also indicative of the flow behavior, with lower values corresponding to better flow. Five replicates were recorded per sample.

Bulk and tapped densities were measured using a 100 mL graduated cylinder. The bulk density (ρ_bulk) was calculated after carefully transferring the powder into the cylinder without tapping. The tapped density (ρ_tapped) was measured after 100 taps using the same device. The following formula was used:

ρ_bulk = m/V_bulk  (g/cm^3^) (1)

ρ_tapped = m/V_tapped  (g/cm^3^) (2)

Bulk and tapped densities were used to calculate the Carr index and Hausner ratio, as indicators of compressibility and flow characteristics [13]. Carr’s Compressibility Index (CI) was calculated as follows:

CI (%) = [(ρ_tapped − ρ_bulk)/ρ_tapped] × 100(3)

A value below 15% indicates good flow, while a value above 25% indicates poor flow.

The Hausner ratio (HR) was calculated as follows:

HR = ρ_tapped/ρ_bulk(4)

Values below 1.25 indicate good flow, while values above 1.40 suggest poor flow due to cohesiveness. All flowability tests were conducted in triplicate (or quintuplicate, where noted), and mean values were used for analysis.

### 2.6. Quantification of NIC, PIR, and HE Flavonoids

Qualitative and quantitative determinations of NIC and PIR in capsule formulations can be performed using a UV-VIS spectrophotometric method, due to the lack of spectral overlaps, its simplicity, speed, and absence of matrix interference. However, for HE analysis, which contains multiple UV-absorbing compounds such as flavonoids, a high-performance liquid chromatography (HPLC) method was necessary to accurately separate and quantify NIC, PIR, and flavonoids.

The spectrophotometric assay of NIC and PIR was carried out using an Agilent Technologies 8453 UV-VIS spectrophotometer (Santa Clara, CA, USA), with spectra recorded in the 190–300 nm range. NIC and PIR exhibited absorption maxima at 288 nm and 205 nm, respectively. At these wavelengths, no spectral interference from HE flavonoids was observed, as these compounds are not soluble in the methanolic HCl solvent used for NIC and PIR analysis.

Standard solutions of NIC and PIR were prepared in 0.1 M methanolic HCl, over the concentration range of 5–40 µg/mL, and calibration curves were constructed accordingly (r^2^ > 0.998). Capsule contents were accurately weighed, dissolved in the same solvent, filtered through a 0.45 µm membrane, and diluted to the required concentration. Each measurement was performed in triplicate. The method was validated as previously described [14].

HPLC was employed to simultaneously identify and quantify NIC, PIR, and HE flavonoids from the combined capsules. For NIC and PIR analysis, capsules were prepared by dissolving 0.1 g of content in 25 mL mobile phase, centrifuged (5000 rpm, 5 min), then diluted 1:1 with the same mobile phase. For flavonoid analysis, samples were prepared by dissolving 0.1 g of capsule content in 25 mL 40% ethanol, followed by centrifugation at 5000 rpm for 5 min and dilution of the supernatant with mobile phase to obtain a 200 µg/mL solution. A 20 µL injection volume was used for all analyses.

Chromatographic separation was carried out on a Shimadzu-20A HPLC system with UV-VIS detection, using an EC/Nucleosil C18 column (5 μm, 100 × 4.6 mm), with the temperature set at 30 °C. NIC and PIR were separated using a mobile phase of acetonitrile, methanol, and phosphate buffer (40:35:25 *v*/*v*/*v*, pH = 7.0) in isocratic elution, a flow rate of 1.5 mL/min, and UV detection at 288 nm and 205 nm, respectively. Flavonoids were quantified using acetonitrile and phosphate buffer (18:82 *v*/*v*, pH = 3.0), 1.0 mL/min flow, with detection at 325 nm.

### 2.7. In Vitro Dissolution Profiling

The dissolution profiles of the previously described capsule formulations (F1–F4) were tested using an Electrolab Dissolution Tester 1 (basket) in 1000 mL of 0.1 N HCl (pH = 1–2) and phosphate buffer (pH = 6.8) at 37 °C with agitation at 100 rpm. Manual sampling was carried out by withdrawing 10 mL aliquots at 5, 10, 15, 20, 30, 45, and 60 min. After each withdrawal, the volume was replaced with a degassed blank medium.

After filtration through a 0.45 μm membrane and appropriate dilution with methanolic 0.1 M HCl, the concentrations of NIC and PIR were determined by UV-VIS spectrophotometry, as detailed in Section 2.6. Five replicates were performed at each time point.

The dissolution kinetic parameters of the active substances were calculated, and the dissolution rate constant was evaluated based on release in the acidic medium. The calculation of the dissolution rate constant (Kd) was performed using the formula:

Kd = (lnC_1_ − lnC_2_)/(t_2_ − t_1_), (min^−1^) (5)

C_1_ and C_2_ are the concentrations of the dissolved substance at t_1_ and t_2_ time points, respectively.

### 2.8. Stability Studies

Forced degradation studies were conducted to evaluate the chemical stability of the F1 capsules under oxidative, thermal, and photolytic stress conditions [27,28]. For oxidative stress, the content of one capsule was dissolved in 5.0 mL of methanolic 0.1 M HCl, followed by an addition of 3.0 mL 5% hydrogen peroxide, and the mixture was stirred for 6 h at 25 °C. For thermal degradation assessment, the capsules were stored in a thermostatic chamber at 60 °C for one month. Photolytic degradation was evaluated by exposing 30 capsules to UV light (254 nm) for 7 days.

After exposure, 0.5 mL of each test solution was diluted to 25 mL with mobile phase, filtered through 0.22 μm membrane filters, and analyzed by HPLC to detect and quantify any degradation products. The quantitative content of NIC and PIR was also evaluated during the first 48 h of photolytic stress and after 24 h of oxidative and thermal stress exposure.

### 2.9. Antioxidant Capacity Assessment

The antioxidant activity evaluated the contribution of HE and the selected capsule formulation (F1) to oxidative stress modulation.

Three complementary assays were used: 2,2-diphenyl-1-picrylhydrazyl (DPPH) radical scavenging method [29], Trolox Equivalent Antioxidant Capacity (TEAC) [30], and ferrozine iron chelation method [31].

For DPPH assay, methanolic solutions of HE and the combined capsule were analyzed spectrophotometrically at 517 nm. Trolox, used as a standard antioxidant, was tested in the concentration range of 1–7.5 µg/mL to generate the calibration curve (R^2^ = 0.9902; y = 7.9021x + 25.366).

The TEAC assay was performed using the ABTS•^+^ decolorization method, with absorbance measured at 734 nm. The ABTS•^+^ solution was prepared by reacting 2 mM ABTS with 70 mM potassium persulfate and diluted to 0.700 ± 0.020. Samples (1 mg/mL) were tested against a Trolox standard curve (2.5–30 µM; R^2^ = 0.9973, y = 2.7269x + 16.587), and results were expressed as μM TEAC/g.

The iron-chelating activity of the samples was assessed by measuring the inhibition of the ferrozine–Fe^2+^ complex formation, based on the decrease in absorbance at 562 nm. EDTA was used as a reference chelator. Results were statistically evaluated (*n* = 6) and expressed as the median (Me) and interquartile range (IQR).

All measurements were performed in triplicate.

### 2.10. Acute Toxicity (AT) Evaluation

The AT of the F1 capsule formulation was evaluated using the fixed dose method, in accordance with OECD Guideline 420 [32]. A total of 54 Swiss albino mice (27 males and 27 females), aged 12 weeks and weighing between 18 and 26 g at the study’s initiation, and 30 Wistar rats (12 males and 18 females), weighing 210–240 g, were used for the study. Animals were randomly assigned to groups of six (*n* = 6) per dose level, while six served as controls. In mice, increasing doses of 100, 500, 1000, and 2000 mg/kg body weight were administered either orally (by gavage) or intraperitoneally. In rats, intraperitoneal test doses of 100, 300, 1000, and 2000 mg/kg were administered.

Animals were housed individually under standard laboratory conditions. Observations included general behavior, spontaneous motor activity, food and water intake, and physical appearance for 7 days post-administration.

### 2.11. Assessment of Ototoxicity (OT) Potential

OT was assessed in 12 male Wistar rats, aged 3–4 months and weighing 180–250 g. Animals were randomly divided into three groups (*n* = 4 per group): group 1 received 0.9% NaCl, group 2 received gentamicin (40 mg/mL i.m.), and group 3 received the combined capsules (F1 formulation, 1000 mg/kg). The capsule content was diluted in fixed volumes of 0.9% NaCl and administered orally, via gavage, in three separate doses during the 14-day study period. All animals were acclimatized under standard laboratory conditions (plastic housing boxes, controlled environment, and regulated feeding and watering). The animals were monitored for 14 days. Observations included general behavior, food and water consumption, spontaneous motor activity, coordination, response to physical stimuli (light, sound), respiratory function, and skin and mucous membranes status. Body weight was recorded on days 1, 7, and 14 prior to euthanasia. Ototoxicity was assessed on day 0 and on day 14 using an otoscopic examination, Preyer’s reflex, tympanometry, transient otoacoustic emissions (TOAE), and distortion product otoacoustic emissions (DPOAE) [33].

### 2.12. Ethical Considerations

The preclinical investigations on animals (AT and OT) were approved by the Research Ethics Committee of IP USMF “Nicolae Testemițanu,” decisions no. 27 from 14 November 2016 and no. 45 from 26 February 2020. The toxicological and pharmacological studies were conducted in the Laboratory of Preclinical and Clinical Evaluation of Medicines at the Scientific Center for Medicines.

## 3. Results and Discussion

### 3.1. Physicochemical Compatibility Results

#### 3.1.1. Differential Scanning Calorimetry (DSC)

**Figure 2:**
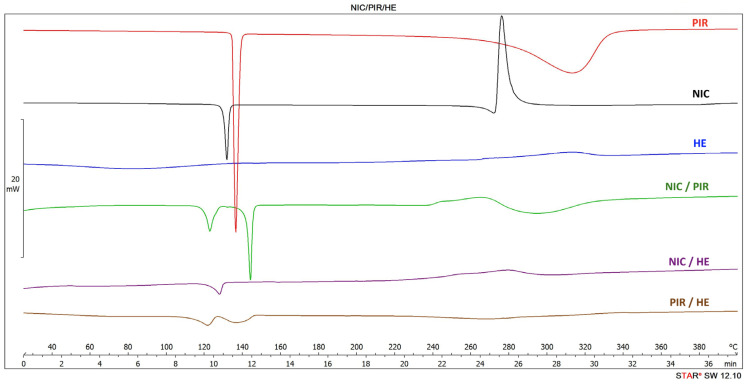
DSC thermograms of NIC, PIR, HE, and their binary physical mixture (1:1, *w*/*w*).

The thermal behavior for NIC, PIR, and HE, along with that of their binary physical mixtures (1:1, *w*/*w*), is presented in Figure 2.

The thermogram for NIC displays the characteristic endothermic melting peak at 132 °C, consistent with values reported in the literature [34,35]. PIR exhibited an endothermic peak at 135 °C. This value is lower than the melting point of the pure compound, typically reported at 152 °C, refs. [36,37], and may be attributed to differences in polymorphic form, crystal structure, or minor impurities.

In the NIC/PIR mixture, the peaks corresponding to the individual substances were retained, suggesting the absence of significant interactions between components. A detailed analysis of the DSC thermogram of HE shows three endothermic peaks, at 148 °C, 184 °C, and 262 °C (see Supplementary file, Figure S1). The assignment of these peaks was challenging due to the complexity of the vegetable matrix, which induces multiple interactions that cannot always be discernible using conventional DSC methods.

Based on the literature data, the peaks at 184 °C and 262 °C were attributed to the melting point of rutoside [38] and vitexin [39], respectively. The peak observed at 148 °C may correspond to vitamin B1, which has a reported melting point at 164 °C [40]. The discrepancy in the melting point may be attributed to matrix complexity or interactions with other constituents in the extract, which can lead to a depression of the thermal signal. Such behavior is not uncommon in multicomponent natural matrices, where compound purity and physicochemical interactions influence thermal profiles [41].

**Figure 3:**
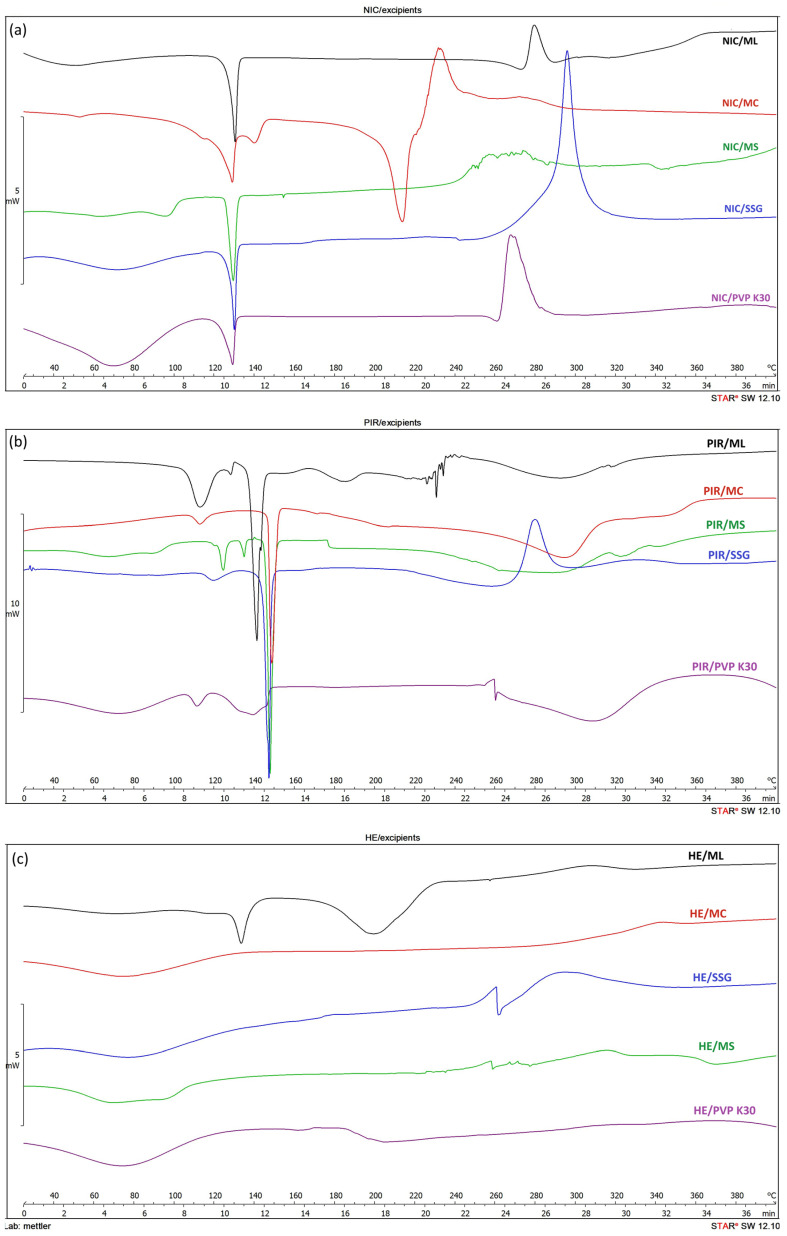
DSC thermograms of binary physical mixtures (1:1, *w*/*w*) of NIC with excipients (**a**), PIR with excipients (**b**), and HE with excipients (**c**).

Upon analyzing the DSC thermograms of binary physical mixtures (1:1, *w*/*w*) of the three main components (NIC, PIR, and HE) with the excipients (Figure 3), differences can be observed compared to the thermal events recorded for the individual components, particularly in the case of PIR and HE mixtures. In the case of PIR mixtures, these differences may be attributed to the polymorphism of PIR. New peaks appear compared to those recorded in the thermograms of the individual components, most likely due to the formation of PIR metastable and stable polymorphic forms during physical mixing with the excipients [36,42].

In the thermograms of the binary mixtures of HE, the thermal events characteristic to the individual components (rutoside, hyperoside) disappeared, which could suggest the possibility of interactions between the components of HE and the other component of the mixture. To further investigate the DSC results, FTIR and XRPD were performed.

#### 3.1.2. Fourier Transform Infrared Spectroscopy (FTIR)

**Figure 4:**
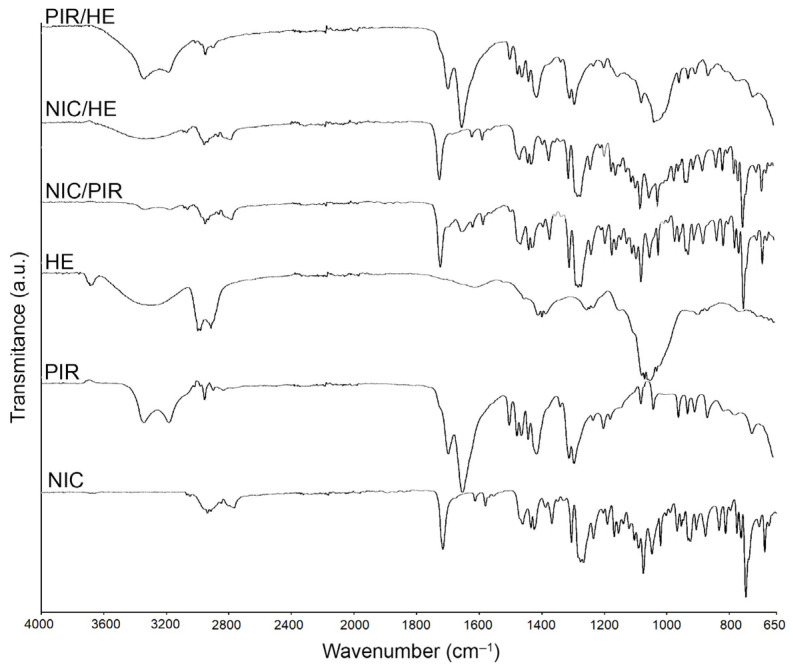
FTIR spectra of NIC, PIR, HE, and their binary physical mixtures (1:1, *w*/*w*), recorded in the range of 4000–650 cm^−1^.

**Figure 5:**
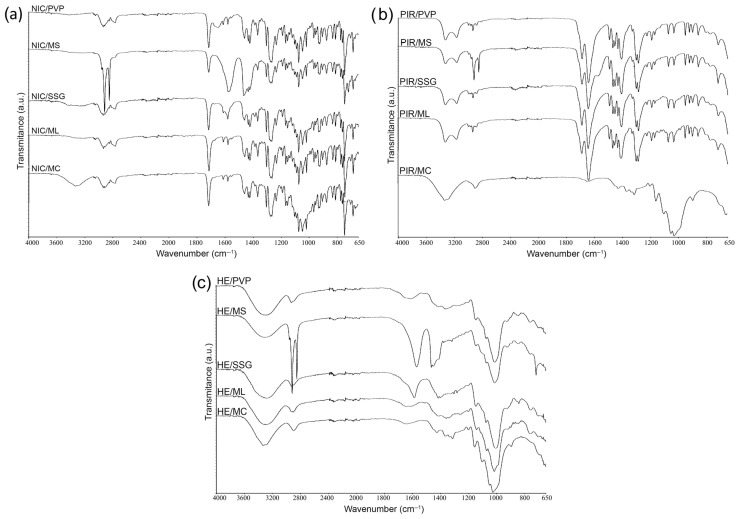
FT-IR spectra of binary physical mixtures (1:1, *w*/*w*) of (**a**) NIC with excipients, (**b**) PIR with excipients, and (**c**) HE with excipients, in the range of 4000–650 cm^−1^.

The FTIR spectra of NIC (Figure 4) display the main functional characteristic groups at 3684.1 cm^−1^ (primary N-H), 1715.6 cm^−1^ (C=O stretching), 1424.2 cm^−1^ (C-H stretching), 1266.4 cm^−1^ (C-N stretching), 1075.5 cm^−1^ (aliphatic C-C), and 762.9 cm^−1^ (ortho distribution), which correlate with the literature data [43,44]. These peaks were all observed in the IR spectra of the NIC + PIR and NIC + HE mixtures, as well as in the binary mixtures of NIC with excipients, which confirms the compatibility of these substances and the absence of chemical interactions between APIs (Figure 4) and APIs with excipients (Figure 5a–c).

The FTIR spectra of PIR exhibit characteristic bands in the 3200–3400 range, with a maximum at 3322.7 cm^−1^ (N-H stretching); at 1687.4 cm^−1^ a band for the carbonyl (C=O stretching); group C-N (stretching) with absorption bands at 1286.5 cm^−1^; band characteristic of stretching vibrations of C-H bonds (C-H stretching) at 2933.3 cm^−1^; maximum at 1643.1 cm^−1^ characteristic for the amide group, all correlating with the literature data [45,46]. Furthermore, all the aforementioned bands were also detected in the spectrum of binary mixtures of PIR with NIC, HE (Figure 4), MC, ML, SSG, MS, and PVP (Figure 5b).

The FTIR spectrum of HE is complex, reflecting various chemical classes such as flavonoids, phenolic acids, proanthocyanidins, and triterpenoids, with multiple overlapping absorption bands corresponding to different functional groups [47,48]. Distinct bands appear for O-H groups at 3284.7 cm^−1^, C=C aromatic groups between 1400 and 1600 cm^−1^, and C-O stretching for alcoholic, ether, or ester groups between 1000 and 1300 cm^−1^. Additional bands from glycosidic structures emerge in the 900–1200 cm^−1^ range, and aliphatic/aromatic C-H bonds at 2900–3000 cm^−1^ (Figure 4). APIs such as NIC and PIR (Figure 4), along with auxiliary substances/excipients, did not alter the spectral behavior of HE (Figure 5c).

#### 3.1.3. Powder X-Ray Diffraction (PXRD)

**Figure 6:**
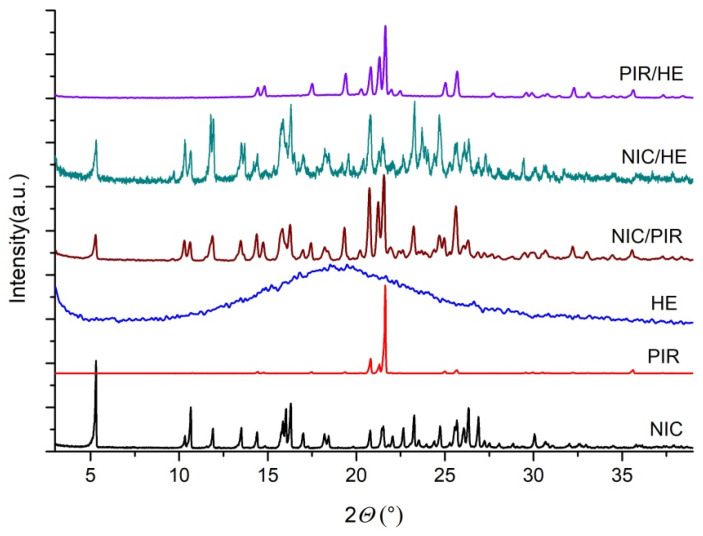
PXRD patterns of NIC, PIR, HE, and their binary physical mixtures (1:1, *w*/*w*).

The PXRD patterns (in the 2θ domain = 3–40°) of NIC, PIR, HE, and their binary physical mixtures (1:1, *w*/*w*) are presented in Figure 6. The diffraction patterns of NIC and PIR showed the presence of numerous distinct peaks, indicating that these substances are crystalline materials. The 2θ values of the more intense peaks of NIC were 5.32; 10.66; 11.91; 13.51; 14.39; 15.86; 16.03; 16.30; 17.01; 18.20; 18.43; 20.77; 21.52; 22.05; 22.66; 23.27; 24.72; 25.67; 26.07; 26.33; 26.89; and 30.06. The 2θ values of the more intense peaks of PIR were 20.81; 21.30; and 21.64. In contrast, HE showed no distinct diffraction peaks, confirming its amorphous nature. No information on the PXRD analysis of HE was found in the literature. Estimating the 2θ values of the HE peaks in the mixture with NIC and PIR is difficult, but an increase in the intensity of the diffraction peaks could be observed at 2θ values between 14.00 and 25.00.

The PXRD patterns of the physical mixtures of NIC with HE, NIC with PIR, and NIC with HE reflected the diffraction profiles of the individual substances, with no new peaks detected, indicating complete compatibility of the analyzed substances.

**Figure 7:**
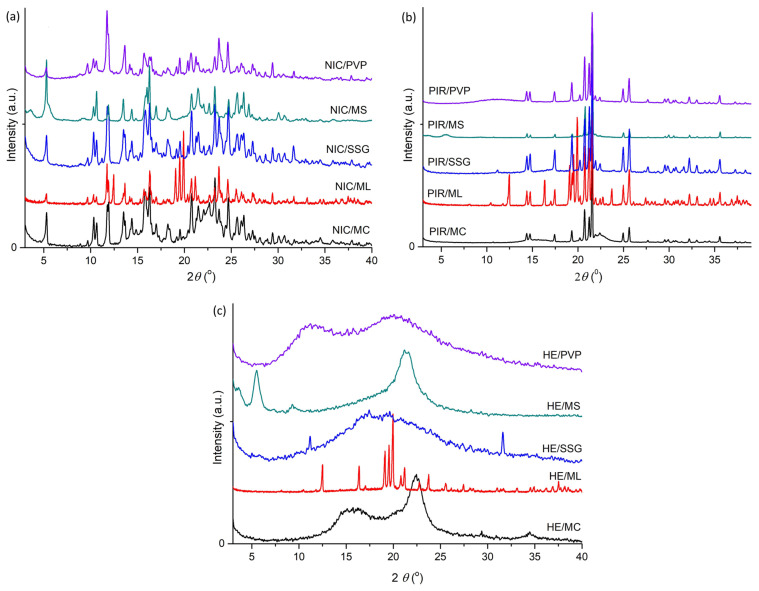
PXRD patterns of binary physical mixtures (1:1 *w*/*w*) of (**a**) NIC with excipients, (**b**) PIR with excipients, and (**c**) HE with excipients.

Similarly, in the PXRD patterns of the binary physical mixtures (1:1, *w*/*w*) of NIC, PIR, and HE with each of the excipients used in the study (MC, ML, SSG, MS, PVP, and MC) (as shown in Figure 7a–c), no changes were observed compared to the diffraction patterns of the individual substances. This indicates the solid-state compatibility of each API with the excipients included in the study.

In conclusion, the DSC, FTIR, and PXRD analyses demonstrated the physicochemical compatibility between the active substances (NIC, PIR, and HE) and excipients. The absence of significant interactions suggests that the stability of the product is unlikely to be compromised by incompatibilities between components [49,50].

### 3.2. Pharmaceutical Formulation Development and Stability Evaluation

#### 3.2.1. Pharmaco-Technological Parameters of Capsule Formulations

Four capsule formulations containing active substances NIC, PIR, and HE were developed (Table 1). The dosages of the active principles were calculated based on their pharmacotherapeutic indications, toxicity profiles, and the anticipated effects.

Following preparation, the formulations were subjected to rheological studies, including the angle of repose, Carr’s index, and Hausner ratio. The results are presented in Table 2.

As presented in Table 2, F2 (MC/MS/PVP) exhibited the poorest flow properties, as indicated by a high tilting angle (45°), a Carr’s index of 30.6%, and a Hausner ratio of 1.44, all suggesting high interparticle cohesion and poor flowability. In contrast, F1 and F3 formulations, both containing monohydrate lactose, demonstrated better flow properties, with Carr’s index values below 13% and Hausner ratios around 1.14–1.15, indicating good flowability and a more uniform dose distribution during the encapsulation [51]. In the presence of active substances, F1 and F4 exhibited moderate Carr’s Index values (16–18%) and suitable Hausner ratios (1.21–1.22), indicating an adequate flow performance. Therefore, the flow of F1 was only slightly affected by the presence of active substances.

#### 3.2.2. Quantification of Active Substances and Flavonoids

For the spectrophotometric assessment of NIC and PIR, the standard solution of NIC showed a well-defined absorbance maximum at 282 nm, while the standard solution of PIR shows a characteristic maximum at 205 nm (see Supplementary file, Figure S2). The determination of NIC and PIR from the combined capsules was carried out across three experimental laboratory series, with six distinct repeated analyses performed. The developed UV-VIS spectrophotometric method was validated, as previously described [14].

**Figure 8:**
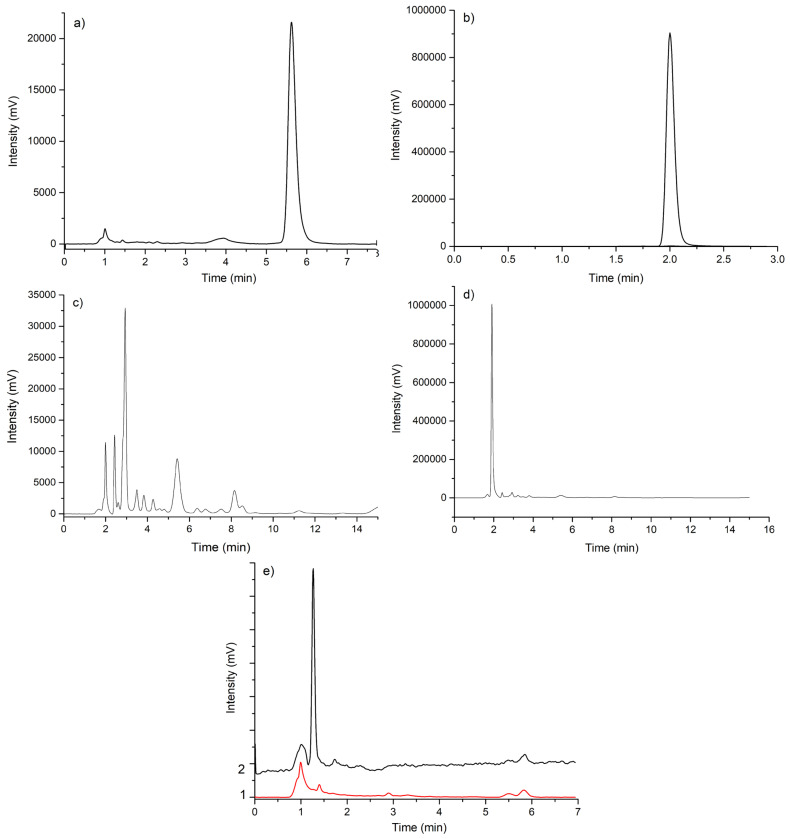
Chromatograms for: (**a**) standard NIC, λ = 282 nm; (**b**) standard PIR, λ = 205 nm; (**c**) sample NIC, λ = 282 nm; (**d**) sample PIR, λ = 205 nm; (**e**) excipients-only at λ = 205 nm (1); and λ = 282 nm (2).

For the HPLC assay, chromatograms of standard solutions with concentrations of 20 μg/mL NIC and 1000 μg/mL PIR were recorded under the previously specified conditions (Figure 8). The retention times were 1960 min for PIR, and 5610 min pentru NIC.

The NIC and PIR content in the pharmaceutical formulations was calculated based on calibration curves.

The developed HPLC-UV method was validated following ICH guidelines [52]. The method demonstrated excellent linearity for PIR (600–1200 µg/mL, r^2^ = 0.9994) and NIC (5–30 µg/mL, r^2^ = 0.9995), with low LOD/LOQ values (PIR: 50.57/153.27 µg/mL; NIC: 0.95/2.88 µg/mL), high precision (RSD < 0.7%), and robustness under deliberate variations in flow rate, column temperature, and mobile phase composition. Accuracy ranged from 99.95% to 100.02% recovery, with RSD values ≤ 0.69%. All the validation parameters are summarized in Supplementary Material Table S1.

The flavonoid concentration was quantified by HPLC using rutoside trihydrate as the external standard (Table 3). The retention times observed for the HE (5.67 min) and capsule extract (5.41 min) were consistent with that of the standard (5.65 min), confirming the identity of the detected flavonoid compounds. These results indicate that the technological process did not alter the flavonoid content of the plant extract.

The flavonoid content per 500 mg capsule ranged between 0.400% and 0.600%. Statistical evaluation of the capsule results, recalculated as rutin equivalents, yielded a mean content of 0.5204%, with a standard deviation (SD) of 0.0083 and interquartile range (IQR) of 0.0165. The results were normally distributed (*p* > 0.05), indicating consistency in flavonoid content across the tested capsules.

#### 3.2.3. Dissolution Profiles of NIC and PIR in Capsule Formulations

**Figure 9:**
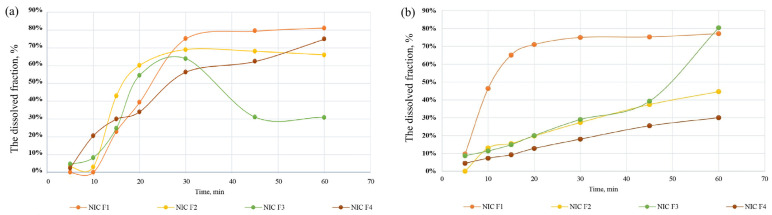
Dissolution profiles of NIC from capsules in acidic (**a**) and neutral (**b**) media.

**Figure 10:**
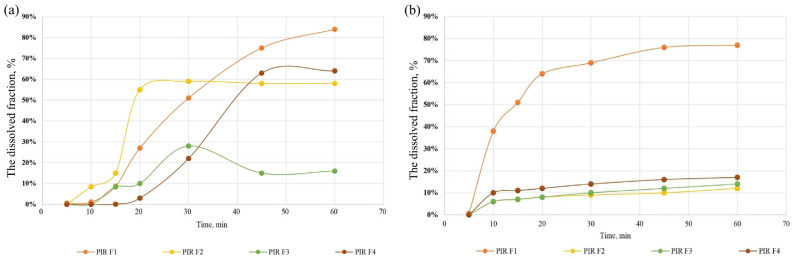
Dissolution profiles of PIR from capsules in acidic (**a**) and neutral (**b**) media.

Dissolution testing was conducted on all four capsule formulations, with a quantitative analysis of NIC and PIR performed spectrophotometrically. The dissolution profiles of NIC and PIR from the combined capsules were evaluated in both acidic and neutral media. Analysis of the dissolution profiles for NIC and PIR in the tested formulations (Figure 9 and Figure 10) revealed that formulation F1 exhibited superior release in the acidic medium for both compounds, with NIC reaching 75% dissolution after 30 min and PIR after 45 min. This suggests superior bioavailability in an acidic environment, which is crucial for rapid absorption in the gastrointestinal tract [53]. In contrast, F2, F3, and F4 showed lower dissolution rates, especially in a neutral medium, where the release was slower and uneven, most likely due to the influence of excipients on the dissolution behavior.

Interestingly, in the acidic medium, formulation F3 demonstrated significantly reduced dissolution rates for both active ingredients, especially beyond the 30-minute mark. This behavior can be attributed to the specific excipient composition of F3. Unlike formulation F1, F3 lacks SSG, a superdisintegrant known to promote rapid capsule disintegration through swelling. In addition, the inclusion of PVP K30 as a binder may contribute to increased viscosity in the diffusion layer, especially under acidic conditions, which could delay drug release [54]. Furthermore, F3 does not contain MC, a commonly used wicking agent that facilitates medium penetration through capillary action. The absence of both MC and SSG likely impaired the wetting, disintegration, and dispersion processes, leading to a slower and less efficient dissolution of NIC and PIR. These results reinforce the importance of selecting appropriate disintegrants and fillers to ensure a rapid and consistent release from capsule formulations.

As the dissolution profiles indicated superior results for F1 and F2 in the acidic medium, the kinetic parameters and dissolution rate constants of the active substances were calculated specifically for these two formulations under an acidic medium (see Supplementary file, Table S2) using Equation (5). The dissolution constant of NIC from formulation F1 was 1.36 times higher, while that of PIR was 1.03 times higher compared to formulation F2. Also, for NIC in an acidic medium, F1 could be considered more efficient for providing a more stable release control, as it exhibits a more uniform and consistent dissolution profile over time. For PIR, both F1 and F2 follow a first-order dissolution process, with relatively constant release behavior, but F1 stands out for a faster release and higher dissolution rate during the test. These results demonstrate that F1 formulation is more advantageous in terms of the dissolution rate and dissolution kinetics.

This superior dissolution behavior can be explained based on the excipient composition. The inclusion of SSG in F1 formulation acts as a superdisintegrant, promoting rapid swelling and capsule breakup. This mechanism enhances surface area exposure and dissolution rate. The inclusion of SSG or other superdesintegrants such as kollidon CL or ludiflash exhibited superior drug release profiles with faster drug release, as seen for clonazepam, which are orally fast disintegrating tablets [55]. While SSG is typically used at concentrations between 2 and 8% *w*/*w* [56], in F1 it is present at a lower level (0.5% *w*/*w*), yet it still appears to enhance dissolution. This may be due to a complementary interaction with ML, the main water-soluble filler in F1, which is known to improve wettability and the dispersion of drug particles. Lactose-containing formulations of diltiazem hydrochloride demonstrated a superior drug release (>99% in 8 h) compared to those with microcrystalline cellulose or no excipients due to lactose’s high-water solubility, which enhances polymer hydration, swelling, and drug diffusion [57]. Based on its favorable rheological behavior and consistent dissolution profile, the F1 formulation was selected as the optimal candidate for further evaluation, including stability assessments and in vivo evaluations.

#### 3.2.4. Stability Studies Under Stress Conditions

The influence of various stress factors, including temperature, oxidation, and light, on the stability of NIC, PIR, and HE in F1 capsule formulation was further investigated. Following forced degradation, the APIs and rutin were quantified using the HPLC method presented earlier. An example of chromatogram obtained after forced degradation is presented in the Supplementary file, Figure S3.

**Figure 11:**
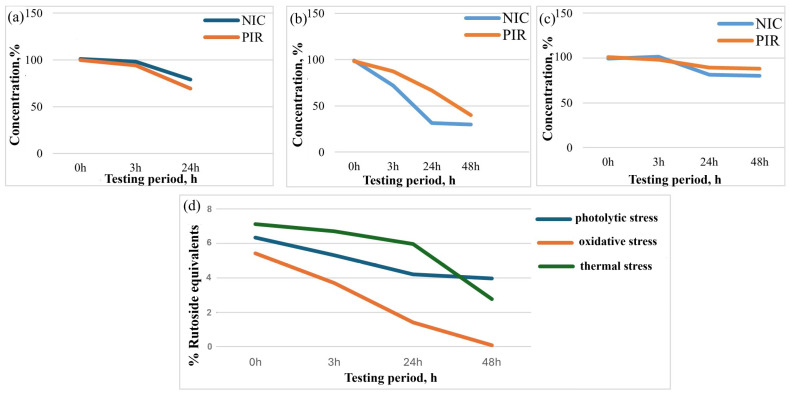
Changes in NIC and PIR concentrations following (**a**) oxidative, (**b**) photolytic, and (**c**) thermal stress. (**d**) Changes in flavonoid content in capsules, expressed as % *m*/*m* rutoside equivalents, following photolytic, oxidative, and thermal stress.

The most significant changes in APIs concentrations occurred under the influence of light and oxidants (Figure 11a–c).

After 24 h of oxidative stress with 5% hydrogen peroxide, NIC degraded by approximately 12%, while PIR degraded by 30%. Light had a negative impact on the substances, with NIC undergoing a rapid degradation of nearly 68% after 24 h of photolytic stress. In the case of PIR, a decrease in concentration of 58% was observed over 48 h. These findings emphasize the importance of protecting the product from light and oxygen to preserve its efficacy [58]. Temperature is also an important degradation factor for both substances, with NIC losing 19.07% and PIR 12.75% of their concentration.

The content of flavonoids, expressed as % rutoside equivalents, was determined after exposure to oxidative, thermal, and photolytic stress conditions of the F1 capsule (Figure 11d). The results demonstrate a significant decrease in flavonoid concentration, with oxidative stress causing the most rapid degradation, followed by thermal stress, while photolytic stress has the least impact on rutin stability.

### 3.3. Evaluation of Antioxidant Activity and Safety

#### 3.3.1. Determination of Antioxidant Activity

The oxidative capacity of the capsules was assessed to verify the preservation of antioxidant properties after formulation and to explore a potential link between these properties and the observed otoprotective effects, given the role of oxidative stress in sensorineural hearing loss.

The antioxidant activity of the dried HE and F1 capsule formulation was determined using the DPPH radical scavenging method and the ABTS•+ cation radical decolorization method. Additionally, the Fe^2+^ chelating capacity was also determined.

Following DPPH assay, HEs exhibited notable antioxidant activity by scavenging 50% of DPPH free radicals at a concentration of 88.43 µg/mL (Table 4). In comparison, the combined capsules achieved the same level at a concentration of 99.16 µg/mL, suggesting a slightly lower antioxidant efficiency. Trolox, used as a reference antioxidant, showed a much lower IC_50_ value, confirming its superior radical scavenging potency.

The results of the ABTS•+ cation radical scavenging assay were consistent with the DPPH findings, indicating that the dry HE has a significantly higher antioxidant activity than the F1 formulation (*p* = 0.003948, Table 4). In parallel, the quantification of flavonoids indicated higher concentrations of rutoside in the dry extract (0.586%, IQR 0.011) compared to the F1 capsules (0.524%, IQR 0.008). Correlation analysis using the non-parametric Spearman test (*p* = 0.547) revealed a direct, though statistically non-significant, relationship between the antioxidant activity of the dried extracts in reducing the ABTS•+ radical cation and the flavonoid content (expressed as rutoside %). This suggests that rutoside, as a representative flavonoid concentration in the sample, may contribute to the observed antioxidant effect.

The assessment of the iron-chelating capacity revealed comparable results between the HE and the combined capsule. The dry extract demonstrated a chelating efficiency of 77.19%, while the combined capsules achieved a slightly lower value of 59.87%. Both samples exhibited statistically significant activity (*p* = 0.000120 and 0.000490, respectively) when compared to the positive control, EDTA (Table 4).

Altogether, these results confirm that the antioxidant activity of the HE is largely retained following incorporation into the capsule formulations. Although minor reductions were observed compared to the dry extract, the formulated capsules still exhibited substantial free radical scavenging capacity, iron-chelating potential, and overall antioxidant efficiency.

Our results fully align with the existing data about the antioxidant potential of HE. This activity, primarily attributed to its rich content of phenolic compounds (chlorogenic acid, procyanidine B2, and epicatechin [59]), polysaccharides, and alkaloids [21], varies depending on the plant part, extraction method [60], and hawthorn variety [61]. Studies show that hawthorn extracts can enhance endogenous antioxidant enzymes (such as superoxide dismuthase, catalase, and glutathione peroxidase) and reduce oxidative damage markers like malondialdehyde [21]. Given these effects, hawthorn is considered a promising natural supplement for preventing oxidative stress-related diseases such neurodegenerative disorders [21] or hearing impairments, which have also been linked to excessive free radical production and oxidative damage.

#### 3.3.2. Determination of Acute Toxicity

In mice, administration of the F1 formulation capsule at a dose of 100 mg/kg, monitored over a period of 7 days, indicated no behavioral changes or mortality. Higher doses, 500, 1000, and 2000 mg/kg, were associated with a temporary period of hypodynamia and a reduced response to exogenous stimuli. Most animals returned to their initial conditions within 4 h following the 500 mg/kg dose, and within 24 h after receiving 1000 or 2000 mg/kg. These results suggest the absence of acute toxicity following both oral and parenteral administration.

In rats, administration of F1 formulation at doses of 300 and 2000 mg/kg similarly caused hypodynamia and a reduced response to exogenous stimuli. All animals returned to their initial state within 24 h for the 300 mg/kg dose, and within 24–48 h for the 2000 mg/kg dose. Necropsy examination of internal organs (tongue, trachea, esophagus, lungs, heart, liver, kidneys, spleen, aorta, urinary bladder, and ovaries) in both experimental and control groups, revealed no visible pathological changes regardless of the administration method. Therefore, the investigated formulation exhibits low toxicity, classified as toxicity class 5 (practically non-toxic) with an estimated LD50 > 2500 mg/kg. Nevertheless, additional studies are necessary to confirm the long-term safety profile.

To our knowledge, there are no studies assessing the acute toxicity of HE in combination with APIs. However, our results are consistent with previous reports on the innocuity of HE alone in animal models. Radi et al. [62] assessed the acute oral toxicity of an aqueous extract from *Crataegus monogyna* leaves and flowers in albino mice. The three treatment groups received 0.5, 1, or 2 g/kg of the extract. Mice were observed for 10 h for immediate signs of toxicity and monitored daily for 14 days. The aqueous extract of *C. monogyna* was found to be non-toxic even at the highest administered dose. The same study also reported antioxidant and antihyperglycemic effects of the extract in rats, without any evidence of acute toxicity [62].

#### 3.3.3. Ototoxicity Studies

Following ototoxicity assessment, no changes in animal behavior were observed across any investigated groups.

In group 1 (control), all auditory parameters were within normal limits: the Preyer reflex (reaction of the laboratory animal to a high-intensity sound stimulus) was present, Type A tympanogram indicated normal tympanic membrane and middle ear function, and otoacoustic emissions were recorded in both ears.

In contrast, in group 2 (gentamicin-induced ototoxicity group), the Preyer reflex and the otoacoustic emissions, including distortion-product otoacoustic emissions, were absent. However, Type A tympanogram indicated a preserved normal function of the tympanic membrane and middle ear. The observed alterations are characteristic for the organ of Corti damage in the inner ear, leading to sensorineural deafness [63].

In group 3 (F1 capsule formulation-treated group), the results mirrored those of the control group: the Preyer reflex was present, the otoacoustic emissions were detected in both ears and the Type A tympanogram was maintained. These results indicate the absence of ototoxic effects from the tested formulation and confirm the proper functioning of the auditory system [64].

To our knowledge, there are no studies assessing the potential of hawthorn extract in sensorineural hearing loss. However, hawthorn (*Crataegus* spp.) has demonstrated several pharmacological effects that could support such a role. These include vasodilatory action on vascular smooth muscle, inhibition of thromboxane A_2_-mediated vasoconstriction and platelet activation, as well as moderate ACE inhibitory [65]. Such effects could enhance cochlear microcirculation and reduce vascular-related inner ear damage, contributing to auditory protection. Additionally, hawthorn exerts strong antioxidant properties, as previously shown, which may help counteract oxidative stress in the cochlea. Furthermore, other species rich in flavonoids, such as *Castanopsis echinocarpa* [66], or Cuscutae Semen and Rehmanniae Radix Preparata [67] have demonstrated protective effects against hearing loss by promoting sensory cell protection and upregulating the expression of genes related to hearing loss or neuronal function.

Also, a study assessing the efficacy and safety of nicergoline combined with oxiracetam (a structural and pharmacological analog of piracetam) in vascular cognitive impairment showed that after 3 months of treatment, all groups showed improved cognitive and daily functioning scores. The combination therapy yielded the most significant benefits, with no notable side effects observed. Importantly, the combination proved more effective than monotherapy without added risk [68].

### 3.4. Limitations of the Study

Despite the encouraging results, the present study has several limitations.

First, although physicochemical compatibility and stability were extensively tested, the bioavailability of the active substances and their potential synergistic interactions in vivo remain to be elucidated. Second, the antioxidant activity was evaluated only in vitro, without investigating the in vivo antioxidant potential of the F1 formulation. Third, safety evaluations were limited to acute toxicity and short-term ototoxicity assessments in small animal models. These models do not always predict long-term toxic effects or rare adverse reactions, nor can they replicate the complexity of the human sensorineural hearing system. Therefore, supplementary studies, including long-term safety evaluations, pharmacokinetic profiling, efficacy testing in disease models, and ultimately clinical trials, are necessary to confirm the therapeutic potential of the developed formulation.

## 4. Conclusions

The present study successfully developed and characterized a novel capsule formulation, containing nicergoline, piracetam, and hawthorn extract, intended for the management of sensorineural hearing loss.

In the first phase, the physicochemical and pharmaco-technical analyses demonstrated the compatibility and stability of the active ingredients and excipients. The proposed formulations were evaluated for flowability, dissolution kinetics in both neutral and acidic media, and stability under oxidative, thermal, and photolytic stress conditions. Following the selection of the optimal formulation (F1), the antioxidant assays indicated that the formulation possesses an important radical scavenging and iron-chelating activity, largely due to the flavonoid content of the hawthorn extract. Finally, acute toxicity studies in mice and rats indicated a high safety margin (LD_50_ > 2500 mg/kg), with no mortality or significant adverse effects. Additionally, ototoxicity assessments revealed no negative impact on auditory function. However, further evaluations are necessary to confirm the long-term safety and efficacy of the proposed capsule formulation.

Taken together, these results support the safety, stability, and functional potential of the novel capsule formulation. Additionally, the study serves as a practical model for the development and evaluation of a newly fixed-dose combination, providing a structured pipeline from preformulation to in vivo safety assessment.

## Figures and Tables

**Table 1:** Combined capsule formulations (amount per capsule).

Component	Role	Formula Code/ Amount Expressed in mg Per Capsule			
		F1	F2	F3	F4
NIC	API	4.5	4.5	4.5	4.5
PIR	API	200	200	200	200
HE	API	50	50	50	50
MS	Lubricant	2.5	2.5	2.0	2.0
MC	Filler/dry binder	-	140.5	-	141.5
SSG	Superdisintegrant	2.5	-	-	2.0
PVP K30	Binder	-	2.5	2.0	-
ML	Filler	240.5	100.0	241.5	100.0
Total		500	500	500	500

**Table 2:** Pharmaceutical–technological parameters, including Carr’s Index, and Hausner ratio. The results are expressed as the mean of three determinations, with *n* = 6 for each formulation.

Formulations	Tilting Angle (°)	Angle of Repose (°)	Initial Bulk Volume, V_0_ (cm^3^)	Tapped Volume, V_t_ (cm^3^)	Initial Bulk Density, d_vi_ (g/cm^3^)	Tapped Density, d_vt_ (g/cm^3^)	Carr’s Index, %	Hausner Ratio
Excipients mixtures								
ML/MS/SSG (F1)	36	23	32	28	0.77	0.88	12.5	1.14
MC/MS/PVP (F2)	45	41	49	34	0.50	0.73	30.6	1.44
ML/MS/PVP (F3)	26	30	31	27	0.73	0.84	12.90	1.15
MC/MS/SSG (F4)	50	48	59	49	0.39	0.47	16.95	1.20
Excipients with active substances								
F1	31	20	30	25	0.33	0.40	16.67	1.21
F2	48	45	35	25	0.29	0.40	28.57	1.38
F3	21	25	30	23	0.33	0.43	23.33	1.30
F4	45	45	55	45	0.18	0.22	18.18	1.22

**Table 3:** Flavonoid concentration in the API, HE, and capsules, expressed as rutoside equivalents.

Sample	Flavonoid Content (% Rutoside Equivalents)	Retention Time (min)
Rutoside trihydrate (standard)	91,8602	5.65
Hawthorn extract	0.58662	5.67
Capsules	0.52041	5.41

**Table 4:** Antioxidant activity of the dried hawthorn extract and combined capsules, determined by DPPH, ABTS, and iron-chelating antioxidant assays.

Samples	DPPH, IC_50_ (µg/mL)	ABTS (μM TE/g Dry Mass)	Iron-Chelating Capacity (%)
Dry hawthorn extract	88.43 (5.581)	20.84 (1.221)	77.19 (0.015)
Combined capsules (F1)	99.16 (2.421)	19.33 (0.349)	59.87 (0.618)
Trolox (reference)	3.7100 (1.84)	-	
EDTA (reference)	-	-	93.54 (0.8475)

Note: All values are expressed as the median (interquartile range) for *n* = 6. IC_50_: half-maximal inhibitory concentration; μM TE/g: micromoles of Trolox equivalents (TE) reported per gram of dry sample.

## Data Availability

The data supporting the results of this study can be obtained from the corresponding authors upon reasonable request.

## Supplementary Materials

The following supporting information can be downloaded at: https://www.mdpi.com/article/10.3390/pharmaceutics17081017/s1, Figure S1: Detailed section of the DSC thermogram for the hawthorn extract (HE), highlighting the thermal events; Figure S2: Absorption spectra of (a) PIR standard, (b) NIC standard, (c) sample from combined capsules, and (d) placebo. In each case, an HCl 0.1 M methanolic solution was used as solvent; Figure S3: The chromatograms obtained for (a) PIR and (b) NIC after exposure to thermal stress (storage in a thermostatic chamber at 60 °C for 30 days); Table S1: Validation results of the HPLC method of NIC and PIR dosage from capsules (average values for the analysis of 3 series of capsules). Table S2: The dissolution kinetics of NIC and PIR in F1 and F2 capsule formulations in acidic medium.
