# Supplementary material for: Development and Preclinical Evaluation of Fixed-Dose Capsules Containing Nicergoline, Piracetam, and Hawthorn Extract for Sensorineural Hearing Loss
Source: Pharmaceutics. 2025 Aug 5;17(8):1017. doi: 10.3390/pharmaceutics17081017 (PMC12389583; doi:10.3390/pharmaceutics17081017)
Supplement: Supplementary file 1 [file pharmaceutics-17-01017-s001.zip › pharmaceutics-3771195-supplementary.pdf]

Supplementary Materials

Development and Preclinical Evaluation of Fixed-Dose Capsules Containing Nicergoline, Piracetam, and Hawthorn Extract for Sensorineural Hearing Loss

Lucia Maria Rus [1], Andrei Uncu [2], Sergiu Parii [2], Alina Uifălean [1], Simona Codruța Hegheș [1], Cristina Adela Iuga [1,3], Ioan Tomuță [4], Ecaterina Mazur [2,5], Diana Șepeli [6], Irina Kacso [7,*], Fliur Macaev [6], Vladimir Valica [2,5] and Livia Uncu [2,5]

1 Department of Pharmaceutical Analysis, Faculty of Pharmacy, "Iuliu Hațieganu" University of Medicine and Pharmacy, 400349 Cluj-Napoca, Romania; lucia.rus@umfcluj.ro (L.M.R.); alina.uifalean@umfcluj.ro (A.U.); cmaier@umfcluj.ro (S.C.H.); cristina.iuga@medfuture.ro (C.A.I.)

2 Scientific Center of Medicines, "Nicolae Testemițanu" State University of Medicine and Pharmacy of Republic of Moldova, MD-2025 Chisinau, Moldova; andreiuncu1990@gmail.com (A.U.); sergiu.parii@usmf.md (S.P.); ecaterina.mazur@usmf.md (E.M.); vladimir.valica@usmf.md (V.V.); livia.uncu@usmf.md (L.U.)

3 Department of Personalized Medicine and Rare Diseases, MEDFUTURE—Institute for Biomedical Research, "Iuliu Hațieganu" University of Medicine and Pharmacy, 400347 Cluj-Napoca, Romania

4 Department of Pharmaceutical Technology and Biopharmacy, Faculty of Pharmacy, "Iuliu Hațieganu" University of Medicine and Pharmacy, 400012 Cluj-Napoca, Romania; tomutaioan@umfcluj.ro

5 Department of Pharmaceutical and Toxicological Chemistry, "Nicolae Testemițanu" State University of Medicine and Pharmacy of Republic of Moldova, MD-2025 Chisinau, Moldova

6 Institute of Chemistry, State University of Moldova, MD-2028 Chisinau, Moldova; dianashepel@mail.ru (D.Ș.); fliur.macaev@ichem.md (F.M.)

7 National Institute for Research and Development of Isotopic and Molecular Technologies, 400293 Cluj-Napoca, Romania

* Correspondence: irina.kacso@itim-cj.ro; Tel.: +40-745645670

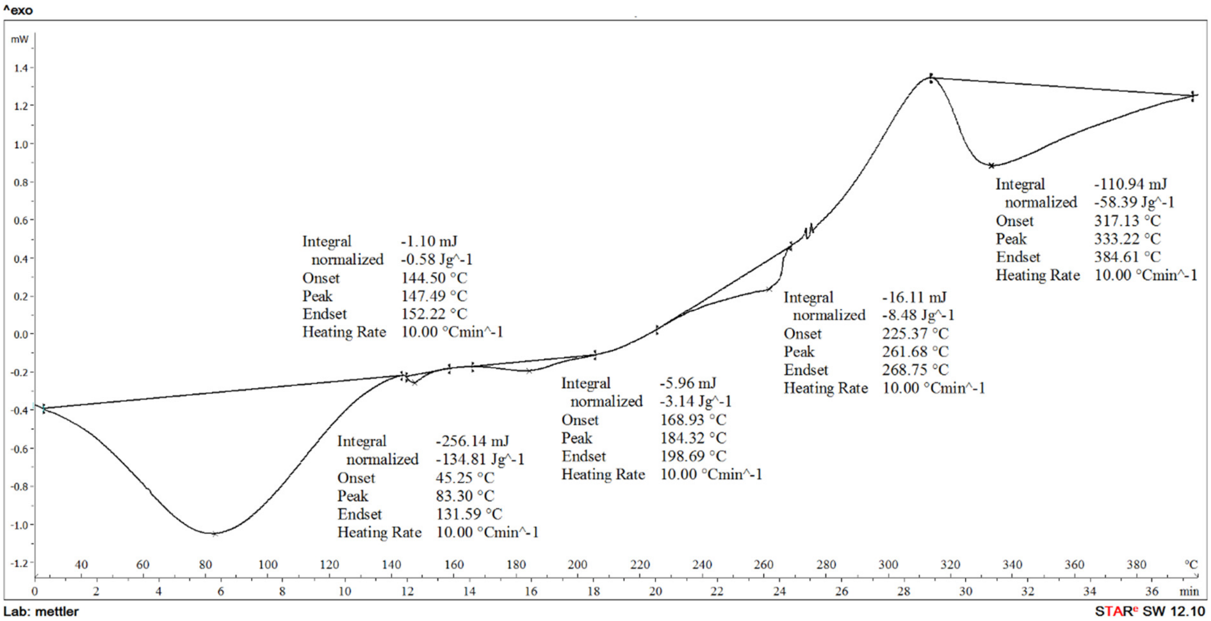

Figure S1. Detailed section of the DSC thermogram for the hawthorn extract (HE), highlighting the thermal events.

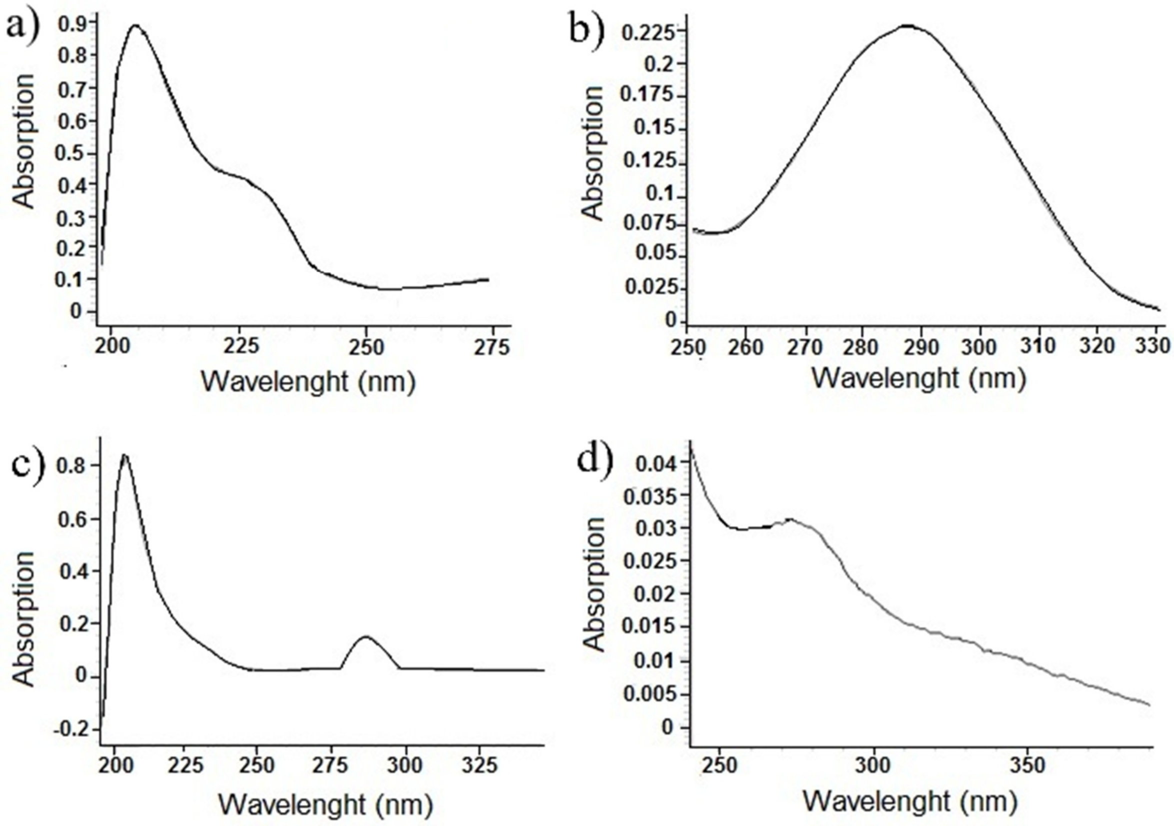

Figure S2. Absorption spectra of a) PIR standard, b) NIC standard, c) sample from combined capsules, d) placebo. In each case, a HCl 0.1 M methanolic solution was used as solvent.

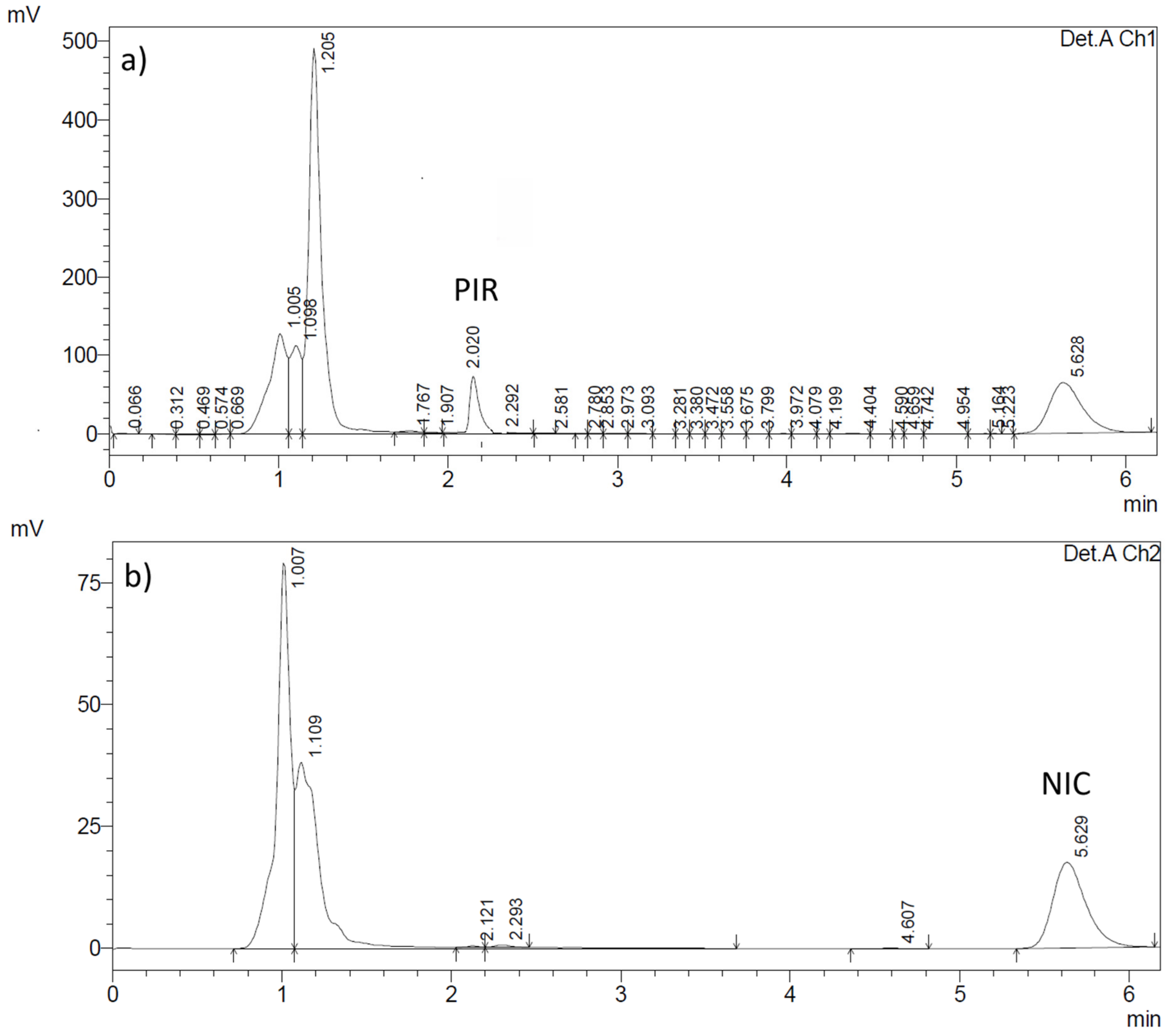

Figure S3. The chromatograms obtained for a) PIR and b) NIC after exposure to thermal stress (storage in a thermostatic chamber at 60 °C for 30 days)

Table S1. Validation results of the HPLC method of NIC and PIR dosage from capsules (average values for the analysis of 3 series of capsules)

API	RT	Lin.	r^2	LOD	LOQ	Equation	Robustness, %RSD			Precision, RSD value, %		Accuracy, % recovery/ %RSD		
							FR ±0.2 mL/min	MP comp.	Col. temp. ±5^0	Repeatability	ID precision	Level 80%	Level 100%	Level 120%
PIR	1.91	600-1200	0.9994	50.57	153.27	y = 4552.8x + 1E+06	0.776	0.951	0.952	0.138	0.578	99.99/ 0.01	100.0/ 0.01	100.0/ 0.01
NIC	5.61	5-30	0.9995	0.95	2.88	y = 62349x - 70291	0.154	0.794	0.793	0.154	0.676	99.95/ 0.69	100.02 /0.01	100.01/0.01

API = Active pharmaceutical ingredient; Col. temp. = Column temperature; FR = Flow rate; ID = Intraday; Lin = Liniarity range (µg/mL); LOD = Limit of detection (µg/mL); LOQ = Limit of quantification (µg/mL); MP comp. = Mobile phase composition (±1.25% acetonitrile and ±1.43% methanol variation); RSD =Relative standard deviation; r^2 = correlation coeficient; RT = Retention time (min).

Table S2. The dissolution kinetics of NIC and PIR in F1 and F2 capsule formulations in acidic medium.

Time	NIC in F1						NIC in F2				
	Q_0, mg	C, %	Q_t, mg	Q_0-Q_t, mg	ln(Q_0-Q_t)	Kd 1, min^{-1}	C, %	Qt, mg	Q_0-Q_t, mg	ln(Q_0-Q_t)	Kd 2, min^{-1}
**5**	4,5	0,00	0	4,50	1,504	0,0000	3,29	0,15	4,35	1,47	0,0041
**10**	4,5	0.002	0,09	4,41	1,484	0,02	3.29	0,15	4,26	1,45	0,1023
**15**	4,5	22.94	1,03	3,47	1,244	0,004	43.21	1,94	2,56	0,94	0,0677
**20**	4,5	39.24	1,77	2,73	1,004	0,048	59.51	2,68	1,82	0,60	0,0262
**30**	4,5	75.01	3,38	1,12	0,113	0,048	68.83	3,10	1,40	0,34	0,0260
**45**	4,5	79.35	3,57	0,93	-0,073	0,0891	68.30	3,07	1,43	0,36	-0,02
**60**	4,5	81.13	3,65	0,85	0,163	-0,016	66.15	2,98	1,52	0,42	-0,06
					**$Kd_{NIC\ F1}$ =**	**0,0322**				**$Kd_{NIC\ F2}$ =**	**0,0237**
**Time**	**PIR in F1**						**PIR in F2**				
	Q_0, mg	C, %	Q_t, mg	Q_0-Q_t, mg	ln(Q_0-Q_t)	Kd 1, min^{-1}	C, %	Qt, mg	Q_0-Q_t, mg	ln(Q_0-Q_t)	Kd 2, min^{-1}
**5**	200	0,00	0	200	5,30		0,00	0	200	5,30	0,0000
**10**	200	1.12	2,24	197,76	5,29	0,002	0.0022	0,0044	199,99	5,30	0,0000
**15**	200	8.68	17,36	182,64	5,208	0,0164	8.46	16,92	183,08	5,21	0,018
**20**	200	26.98	53,96	146,04	4,984	0,0448	14.74	29,48	170,52	5,14	0,014
**30**	200	50.95	101,9	98,1	4,586	0,0398	55.32	110,64	89,36	4,49	0,065
**45**	200	75.09	150,18	49,82	3,908	0,0452	59.24	118,48	81,52	4,40	0,006
**60**	200	84.39	168,78	31,22	3,441	0,0311	58.19	116,38	83,62	4,43	-0,03
					**$Kd_{PIR\ F1}$=**	**0,01495**				**$Kd_{PIR\ F2}$=**	**0,0146**

Q_0, mg = Initial quantity of the active substance (in mg) in the capsule.
C, % = Concentration of the active substance dissolved in the medium at a specific time, expressed as a percentage.
Qt, mg = Quantity of the active substance dissolved at time t, in mg.
Kd, min^{-1} = Dissolution rate constant, in $minutes^{-1}$
